# MicroRNA-34a functions as an anti-metastatic microRNA and suppresses angiogenesis in bladder cancer by directly targeting CD44

**DOI:** 10.1186/s13046-014-0115-4

**Published:** 2014-12-31

**Authors:** Gan Yu, Weimin Yao, Wei Xiao, Heng Li, Hua Xu, Bin Lang

**Affiliations:** School of Health Sciences, Macao Polytechnic Institute, Macao, China; Department of Urology, Tongji Hospital, Tongji Medical College, Huazhong University of Science and Technology, Wuhan, 430030 China; Institute of Urology, Tongji Hospital, Tongji Medical College, Huzhong University of Science and Technology, Wuhan, 430030 China

**Keywords:** Bladder cancer cell, miR-34a, CD44, Metastatic, Angiogenesis

## Abstract

**Background:**

Metastasis have considered as an important clinical obstacle in the treatment of human cancer including bladder cancer. Post-transcriptional regulation has emerged as robust effectors of metastasis. MiRNAs are involved in cancer development and progression, acting as tumor suppressors or oncogenes. In this study, we focus on it that microRNA-34a functions as an anti-metastatic microRNA and suppress angiogenesis in bladder cancer by directly targeting CD44.

**Methods:**

The expression of mir-34a was detected by quantitative real-time PCR. Oligonucleotide and lentivirus were used to overexpress miR-34a. Tube formation assay and transwell assay were used to examine the effect on bladder cancer tube formation, migration and invasion in vitro. Animal models were used to examine the effect on metastasis and angiogenesis in vivo. Luciferase assay was carried out to verify the precise target of miR-34a.

**Results:**

We not only proved that mir-34a was significantly downregulated in bladder cancer tissues and cell lines but also that circulating miR-34a levels are reduced in bladder cancer, and their levels were positively relevance. Gain-of-function experiments investigated that increased mir-34a expression suppressed tube formation and reduced cell migration and invasion. In vivo metastasis, assays also demonstrated that overexpression of mir34a markedly inhibited bladder cancer metastasis. CD31, an endothelial cell–specific marker which stained in T24 tumors to evaluate for blood vessel density, the immunohistochemistry results showed that blood vessel quantification reduced dramatically in the T24 tumors over-expressing mir-34a. Combining with our previous studies and bioinformatics analysis, we expected that CD44 gene was a direct target of mir-34a, siRNA-mediated knockdown of CD44 partially phenocopied mir-34a overexpression suggesting that the pro-apoptotic role of mir-34a may be mediated primarily through CD44 regulation, whereas restoring the expression of CD44 attenuated the function of mir-34a in bladder cancer cells. Additionally, we identified that EMT (epithelial-mesenchymal transition) related proteins could be regulated by mir-34a which indicated that mir-34a could partially reserve EMT.

**Conclusion:**

Our study defines a major metastasis and angiogenesis suppressive role for mir-34a, a microRNA functions as a tumor suppressor in bladder cancer by directly targeting CD44, which would be helpful as a therapeutic approach to block bladder cancer metastasis.

## Background

Bladder cancer is the most common urinary tract malignancy and the fifth most common malignancy in the developed world and is the most common urological tumor in China. This kind of urinary cancer is also with a complicated, multifactorial etiology, involved with both genetic and environmental factors. Besides, the disease is characterized by frequent recurrences and poor clinical outcome when tumors progress to invasive disease. At diagnosis, the most prevalent histopathologic type of bladder cancer in Western countries is transitional cell carcinoma (TCC) accounting for up to 95% of all cases. Approximately 70% of bladder cancer presents with non-muscle invasive bladder cancer tumors, while the remaining cases have invasive tumors [1]. For the patients with non-muscle invasive tumors, they should often be treated by transurethral resection, and with up to 70% of these cases developing at least one recurrence within 5 years [2]. Because of the characters of this disease, early diagnosis and evolution has become very important. Up to now, many studies have investigated molecular biomarkers for prediction of risk and recurrence of bladder cancer. Although some positive results have been obtained and successfully replicated in our previous studies [3-6], they could not explain all the portions of the pathogenesis of bladder cancer and did not put forward the feasible treatment scheme, so the biological theory of bladder cancer need further research.

MicroRNAs are single strand, noncoding RNA molecules that normally functions as negative regulators of mRNA expression of the target genes at the posttranscriptional level [7], through specific targeting of multicellular eukaryotic miR3-UTRs miRs down-regulate gene expression by inducing the degradation or impairing the translation of target mRNAs [8]. Previous accumulated evidence has shown that biological processes such as angiogenic signaling, organ development, cell proliferation, apoptosis avoidance, EMT and tumor invasion pathways are regulated by different microRNAs [9-11]. The current estimate is that >90% of human genes could be controlled by the microRNAs [8]. Deregulation of miRNA expression has been identified in many kinds of human cancer including bladder cancer, and much proved evidences have indicated that some special microRNAs could be functioned as oncogenes or tumor suppressor genes. This crucial findings support us with new strategies in treating human cancer by inactivating oncogenic microRNAs or restoring tumor suppressor microRNAs [12,13]. Furthermore, the first report of altered miRNA expression in bladder cancer appeared in 2007 and detected upregulation of 10 miRNAs [14] and urinary miRNAs have been shown to be clinically useful for noninvasive bladder cancer diagnostics [15]. Recently, it’s reported that miR-19a could act as an oncogenic microRNA in bladder cancer by targeting PTEN when miR-320c could inhibit tumorous behaviors of bladder cancer by targeting CDK6 and both of them could be potential biomarkers of bladder cancer [16,17]. Otherwise, circulating miRNAs have potential use as predictive biomarkers for diagnosis and prognosis of cancer [18]. However, most work has focused on tissue and cell expression with few reports of circulating miRNAs in bladder cancer. Available biomarkers for bladder cancer have limited sensitivity and specificity, and with the urgent treatment in bladder cancer, new markers are needed. Owing to miRNAs’ ubiquity and the characters described above, they also constitute a promising class of biomarkers tailored for human cancer detection, diagnosis, assessment of prognosis, and prediction of response to therapy. Moreover, due to their dynamic and reversible properties, those alterations represent a target for miRNAs-directed therapies.

MiR-34a has been described as a “star” miRNA in cancer research, which commonly functions as a tumor suppressor and is down-regulated in many human cancers [19], Furthermore, the aberrant miR-34a expression has been linked to chemotherapy resistance in a variety of cancer [20-25]. On the other hand, miR-34a is up-regulated in some specific type of cancer, such as papillary thyroid carcinoma (PTC) [26]. Abnormal proliferation and apoptosis are main characters of cancer cells. In PCT, miR-34a promotes proliferation and suppresses apoptosis by targeting Growth arrest specific1 (GAS1), which can suppressor that inhibits cancer cell proliferation and induces apoptosis through inhibition of RET receptor tyrosine kinase, via PI3K/Akt/Bad pathway [26]. However, despite the fact that more and more evidences indicated mir-34a plays a crucial role in the biology processes of bladder cancer, the role of mir-34a in the development and progression remains elucidated in bladder cancer. A better understanding of the role of miR-34a as a potential therapeutic target in bladder genetic analyses suggested that bladder is a genetic disease involving multiple types of gene changes, including miRNAs by the recent decades of research [27,28]. CD44 is a surface adhesion molecule and it has been identified as the prostate cancer and cystic cancer stem cell marker [29,30], and emerged as an important gene in multiple aspects of cancer development [31] and progression [32-34]. The analysis of CD44 isoforms in invasive bladder cancer cells CD44 is largely divided into CD44 standard isoform and variant isoforms, exclusively expressed in mesenchymal cells and epithelial cells, respectively [35-37]. Besides that, CD44v6 could interact with c-Met, thereby enabling cancer cells to be susceptible for growth factor microenvironment, in parallel with EpCAM expression [38]. In fact, the relationship between mir-34a and CD44 may have more connotations. Mutation of p53 can be detected in a majority of cancer and associated with certain critical features of malignancy, including metabolic reprogramming known as Warburg effect [39]. It was reported that p53 had a miR-34a-dependent integrated mechanism to regulate glucose metabolism [39]. Aberrant miR-34a expression has been linked to chemotherapy resistance in a variety of cancer. CD44v8-10 can protect cancer cells from redox stress and up-regulation of CD44v8-10 after chemotherapy has been reported in Li-Fraumeni syndrome (LFS) associated simultaneous osteosarcoma and liver cancer, which is p53-deficinet [40,41]. EMT alters CD44 splicing pattern, in which CD44v is replaced by CD44s. It has also been reported that increased invasion capacity to be due to increased Akt signal pathway. In addition, CD44v and E-cadherin expression are maintained or enriched at the invasive front depending on cancer types [41,42]. However, the function of CD44 in bladder cancer has not yet been fully clarified. Here, we identified mir-34a as down-regulated in bladder cancer, and verified that microRNA-34a functions as an anti-metastatic microRNA and suppresses angiogenesis in bladder cancer by directly targeting CD44.

## Methods

### Patients and samples

In this study, bladder cancer tissue and adjacent normal tissue specimens were collected from patients who were diagnosed histopathologically with bladder cancer and received radical cystectomy in Tongji Hospital, Tongji Medical College, Huazhong University of Science and Technology. All the samples were immediately snap frozen and stored in liquid nitrogen (−180°C). To obtain homogeneous and histological well-characterized samples for RNA analyses, the nature of the tissue and its specified composition were determined by an experienced pathologist. The study was approved by the Institutional Review Board of Huazhong University of Science and Technology, Tongji Medical College, Tongji Hospital, and written informed consent was obtained from all patients.

### Cell culture and transfection

Human bladder cancer cell lines (5637, T24, HT-1376, J82, SCABER and EJ) were obtained from the American Type Culture Collection (Manassas, VA), 5637 and T24 were maintained in RPMI-1640 medium; J82 and SCABER were maintained in DMEM medium; HT1376 was maintained in MEM medium; SV-HUC-1 was also purchased from ATCC and maintained in DMEM/F-12 medium. Cells were cultured and supplemented with 10% fetal bovine serum (FBS) in a humidified atmosphere of 5% CO2 maintained at 37°C. Six synthetic, chemically modified short single or double stranded RNA oligonucleotides (miR-34a mimics, mimics NC, miR-34a inhibitor, inhibitor NC, agomir-miR-34a and agomir-NC) were purchased from Ribo Biotech (Guangzhou, China). Agomir-miRNA is a chemically modified miRNA mimics conjugated with cholesterol. Prevalidated siRNA specific for CD44 and a nonsilencing siRNA control were purchased from Genepharma Biotech (Shanghai, China). Lenti-miR-34a and Lenti-NC were purchased from Genechem Biotech (Shanghai, China). Primary antibody CD44 (1:1000) and GAPDH (1:10000) were purchased from Sigma-Aldrich, St. Louis, MO. Oligonucleotide and plasmid transfection was made by using X-treme GENE siRNA Transfection Reagent (Roche) and FuGene HD Transfection Reagent (Roche) respectively, according to the manufacturers’ protocol. The lentivirus mediating the overexpression of miR-34a and the control vector were supplied by JIKAI Company Shanghai China. Three days after infection, puromycin was added to the media at 2 μg/ml, and cell populations were selected for 2 weeks. Bladder cancer cell line 5637 and T24 were infected at an MOI of 10–20 and harvested 48–72 h post-infection.

### RNA extraction and quantitative real-time RT-PCR

Total RNA was extracted from bladder cancer cell using TRIzol reagent (Invitrogen, Carlsbad, CA, USA) according to the manufacturer’s protocol. The RNA integrity was evaluated by Nano Drop ND-1000 spectrophotometer. Total RNA was extracted from bladder cancer cell using TRIzol reagent (Invitrogen Life Technologies) and then reverse-transcribed using Fermentas RT reagent Kit (Perfect Real Time) according to the manufacturer’s instructions. CD44 expression in bladder cancer cell was measured by qPCR using SYBR Premix Ex Taq on MX3000 instrument. 2 μg of total RNA was converted to cDNA according to the manufacturer’s protocol. PCR was performed in a total reaction volume of 25.0 μl, including 10 μl SYBR Premix Ex Taq(2x), 1 μl of PCR Forward Primer (10 μM), 1 μl of PCR Reverse Primer (10 μM), 0.5 μl ROX Reference Dye II(50x)*3, 2 μl of cDNA, 8 μl of double-distilled water. The quantitative real-time PCR reaction was set at an initial denaturation step of 10 min at 95°C; and 95°C (5 seconds), 63°C (30 seconds), 72°C (30 seconds) in a total 40 cycles with a final extension step at 72°C for 5 min. All experiments were done in triplicate. All samples were normalized to GAPDH. Mir-34a expression in bladder cancer was measured by qPCR using SYBR Premix Ex Taq on MX3000 instrument. 2 ug of total RNA was converted to cDNA according to the manufacturer’s protocol. PCR was performed in a total reaction volume of 20.0 μl, including 9 μl SYBR Premix Ex Taq(2x), 2 μl of PCR Forward Primer (10 μM), 2 ul of PCR Reverse Primer (10 μM), 0.5 μl ROX Reference Dye II(50x)*3, 2 μl of cDNA, 5 μl of double-distilled water. The quantitative real-time PCR reaction was set at an initial denaturation step of 10 min at 95°C; and 95°C (5 seconds), 60°C (20 seconds), 72°C (20 seconds) in a total 40 cycles with a final extension step at 72°C for 5 min. All experiments were done in triplicate. All samples were normalized to U6. The median in each triplicate was used to calculate relative CD44/mir-34a concentrations (ΔCt = Ct median CD44/mir-34a-Ct median GAPDH/U6). Expression fold changes were calculated using 2-ΔΔCt methods. The CD44/mir-34a expression differences between cancer and control were analyzed using Student’s t test within SPSS (Version 17.0 SPSS Inc.). A value of p < 0.05 was considered as statistically significant.

### Transwell assay

Migration and invasion assays were performed as described as follows, bladder cancer cell lines 5637 and T24 were transfected with pre-miR-NC and has-miR-34a mimics for 48h. Transwell inserts that have 6.5-mm polycarbonate membranes with pores 8.0 μm in size (Corning, New York, NY, and USA). Matrigel invasion assay was done using membranes coated with Matrigel matrix (BD Science, Sparks, MD, USA). A density about 1 × 10^5 of 5637 cells or 1 × 10^4 of T24 was suspended and then seeded in the upper chambers of 24-well transwell plates with FBS-free medium. Culture medium containing 10% fetal bovine serum was deposited in the lower chambers. After 12–18 hours cells that migrated were stained by 0.5% crystal violet solution for 15 min and counted. For invasion, transwell membranes were prepared with matrigel for plating infected cells. After 24 hours cells that migrated were stained by 0.5% crystal violet solution for 15 min and counted. Each experiment was performed in duplicate.

### Western blot

Briefly, protein was extracted from cells, Protein extraction with NP40 and were separated on 10% SDS-PAGE gel and then transferred to PVDF membranes (Bio-Rad). Nonspecific binding was blocked by incubating the PVDF membranes with 5% nonfat milk containing 0.1% Tween-20 and then for 2 hours at room temperature. The membrane was incubated Primary antibodies included CD44 (1:1000 dilution; Abcam), E-cadherin,vimentin, N-cadherin, beta-catenin and GAPDH (1:2000 dilution; Cell Signaling Technology) in TBST +5% nonfat milk at 4°C overnight. After washing with TBST, the PVDF membranes were incubated with horseradish peroxidase-conjugated sheep anti-mouse IgG(1:4,000) for 1 hr at 37°C. At last the proteins were visualized using ECL-plus detection system (Pierce).

### Experimental lung and liver metastasis model

The anti-metastatic activity of miR-34a was tested in nude mice T24 lung metastasis model as described previously [25]. T24 cell was stably infected with Lenti-miR-34a or Lenti-NC containing GFP label. Treated cells (2 × 106) were suspended in 100 μL of PBS and injected intravenously via the tail vein. Mice were sacrificed and lungs were resected 30 days later after injection. The incidence and volume of metastases were estimated by imaging of mice for bioluminescence using the Living Image software (Xenogen, Baltimore, MD). The photon emission level was used to assess the relative tumor burden in the mice lungs. Besides that, the mice were killed, their livers were dissected, and tumor nodules were counted under a stereomicroscope (Olympus). Nude mice were manipulated and cared according to NIH Animal Care and Use Committee guidelines in the Experiment Animal Center of the Tongji medical collage of Huazhong University of science and technology (Wuhan, Hubei, Province, P.R.C.)

### In vitro tube formation assay

HUVECs were maintained in endothelial basic medium containing 2% FBS and 1% penicillin/streptomycin or the indicated conditioned media (CM) of 5637 and T24 cells. HUVECs (3 × 10^4) were seeded into a 96-well culture plate pre-coated with Matrigel (BD Biosciences) overnight and then cultured in the indicated condition. After 4–6 h incubation, the formation of tubes was photographed with a phase contrast microscopy (100× magnification, Olympus Instruments, Inc.), and quantified by counting branch points in five randomly selected microscope fields per well. The experiments were conducted twice.

### Dual luciferase assay

To verify the precise target of miRNAs, dual luciferase assays were carried out in both 5637 and T24 cells. Cells (1 × 10^5) were plated in 12-well plates, 24 h before transfection. 50 nmoles of miR-34a mimics/inhibitor or adjacent negative control and 50 ng pmirGLO-30-UTR vector were co-transfected into cells using X-treme GENE transfection reagent (Roche Applied Science). Renilla and firefly luciferase activities were measured with the Dual-Luciferase Reporter system (Promega) 24 hours after transfection. Firefly luciferase activity was normalized to Renilla luciferase expression for each sample. Each experiment was performed in triplicate.

### Statistical analysis

Data from all experiments were presented as means ± s.e.m. from at least three independent experiments and analyzed by Student’s t-test unless otherwise specified (Pearson’s correlation). The miR-34a expression was analyzed by Mann–Whitney test, Wilcoxon matched pairs test for paired data. The correlation between miR-34a level and CD44 protein expression was calculated by Spearman’s correlation. The correlation between miR-34a level in bloodstream and tissues was also calculated by Spearman’s correlation. P-value < 0.05 was considered as statistically significant.

## Result

### Mir-34a expression’s loss in human bladder cancer and circulating mir-34a level was reduced in human bladder cancer

**Figure 1:**
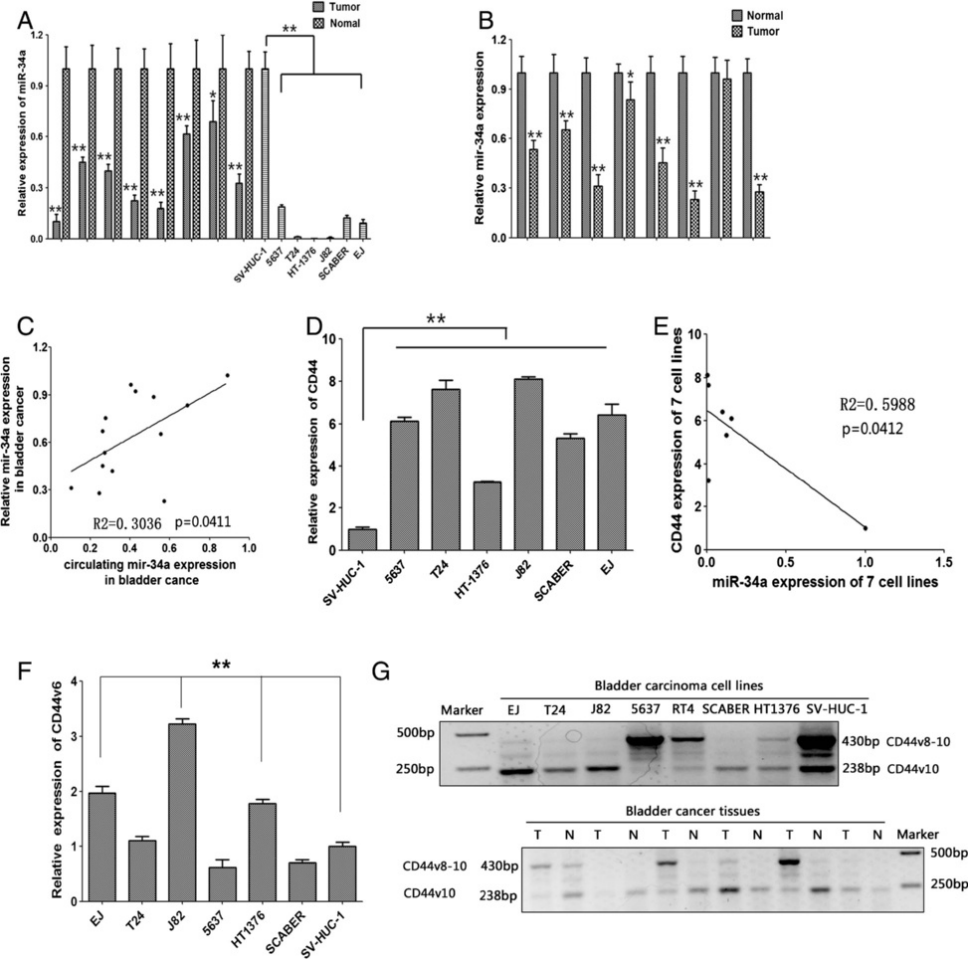
**mir-34a was downregulated in bladder cancer and circulatingmiR-34a levels are reduced in bladder cancer. A**, relative expression of mir-34a expression levels were evaluated by qPCR in bladder cancer.U6 small nuclear RNA was used as an internal control. **B**, relative expression of circulating mir-34a expression levels were evaluated by qPCR in bladder cancer. U6 small nuclear RNA was used as an internal control. **C**, The expression of mir-34a in blood stream positively correlates with the expression in bladder tissues (R2 = 0.3036 P = 0.0411, Pearson’s correlation). **D**, relative expression of CD44 expression levels were evaluated by qPCR in bladder carcinoma cell lines. **C**, miR-34a expression after mimics transcription was detected by qPCR. **E**, MiR-34a inversely correlates with CD44 expression (R2 = 0.5988 P = 0.0412, Pearson’s correlation). **F**, relative expression of CD44v6 expression levels were evaluated by qPCR in bladder carcinoma cell lines. **G**, CD44v expression pattern in human bladder cancer cell lines and bladder cancer tissues. Data are plotted as the mean ± SEM of 3 independent experiments. **, P < 0.01.

To investigate the role of miR-34a in bladder cancer, we firstly evaluated the expression of miR-34a in five bladder carcinoma cell lines (5637, T24, HT-1376, J82, SCABER and EJ) and a non-tumorigenic bladder cell line SV-HUC-1 by qPCR. Compared with SV-HUC-1 cells, these six bladder carcinoma cell lines had a significantly lower level of miR-34a expression, meanwhile, we evaluated the expression of miR-34a in bladder tissues, and we found that mir-34a was downregulated in bladder tissues, compared with the corresponding adjacent control, (Figure 1A, P < 0.01). Besides that, we evaluated the expression of CD44 in bladder cancer cell lines and found the expression of CD44 was up-regulated in these cell lines (Figure 1D, p < 0.01), suggesting a negative correlation between the expression of CD44 and miR-34a in bladder cancer cell lines, which was shown in Figure 1E (R^2^ = 0.5988 p = 0.0412). Additionally, we found that significantly reduced expression of circulating mir-34a in bladder cancer (Figure 1B, p < 0.01), and next, we compared the expression of mir-34a in bladder cancer tissues and bloodstream, showing a positive correlation between them (Figure 1C, R2 = 0.3036 p = 0.0411). Moreover, the data of CD44v expression pattern in human bladder cancer cell lines and bladder cancer tissues were shown in Figure 1F and Figure 1G. All the results above indicated that mir-34a takes a great role in the recurrence and progression in bladder cancer and this may have future application as part of a biomarker profile in bladder cancer.

### Mir-34a overexpression inhibits bladder cancer cell invasion and metastasis by modulating EMT related proteins in *vivo and vitro*

**Figure 2:**
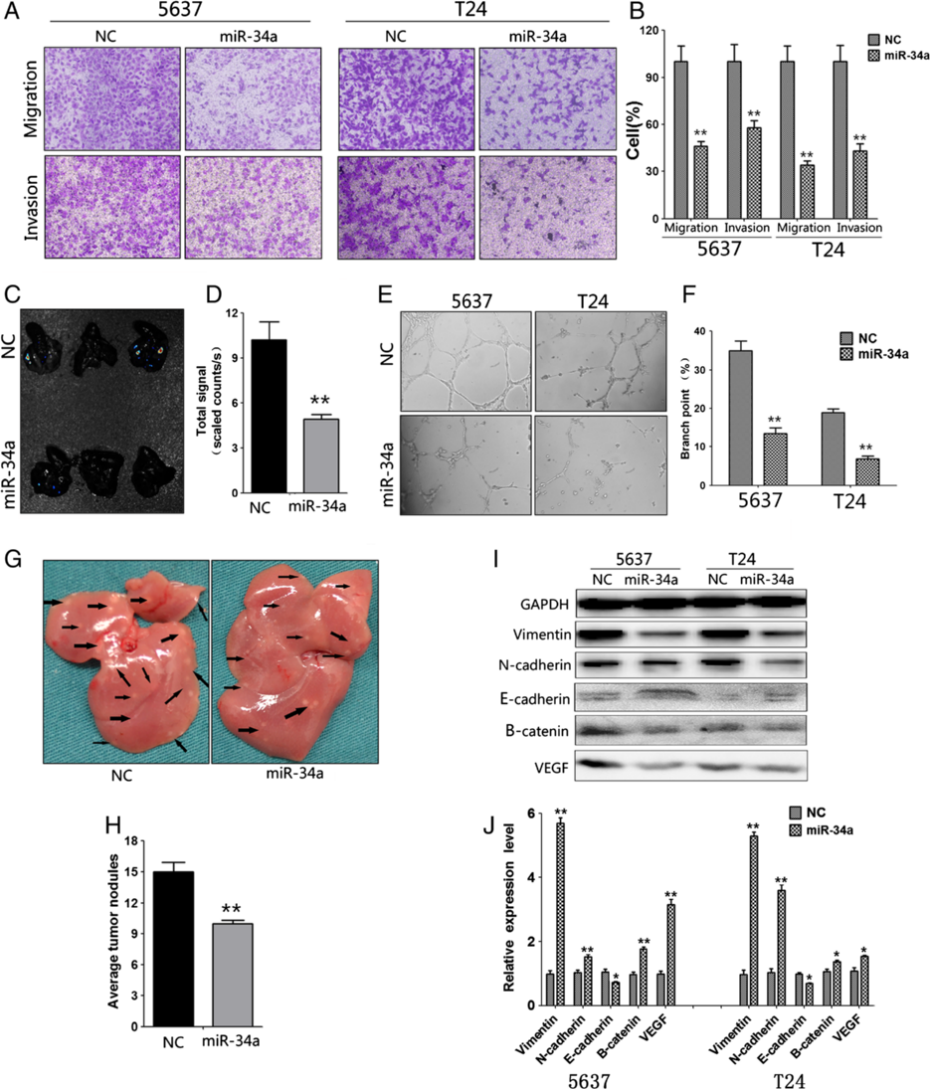
**Overexpression of mir-34a inhibits bladder cancer cell invasion and migration and tube formation. A**, migration and invasion assay for renal cancer cells. Representative photographs were taken at × 200 magnification. **B**, number of migrated and invaded cells were quantified in 4 random images from each treatment group. Results are the mean ± SEM from 2 independent experiments plotted as percent (%) migrating and invading cells relative to NC treatment. **C**, representative bioluminescent images of lungs in nude mice at the 30th days after IV injection. **D**, quantification analysis of fluorescence signal from captured bioluminescence images. **E**, Tube formation of HUVECs was determined by assaying the numbers of branch nodes after 6 h of culture under a phase contrast microscope. **F**, number of branch point was quantified. HUVECs were cultured in the following media: Culture Media of 5637 and T24 cells transfected with mir-34a and NC. **G**, metastatic nodules in the liver. **H**, number of nodules was quantified. **I**, EMT related proteins and VEGF were determined by immunoblot analysis. **J**, the density of blots was quantified to show the expression level of EMT related proteins and VEGF, GAPDH was used as control. NC, Negative Control. ^*^, P < 0.05; ^**^, P < 0.01.

**Figure 3:**
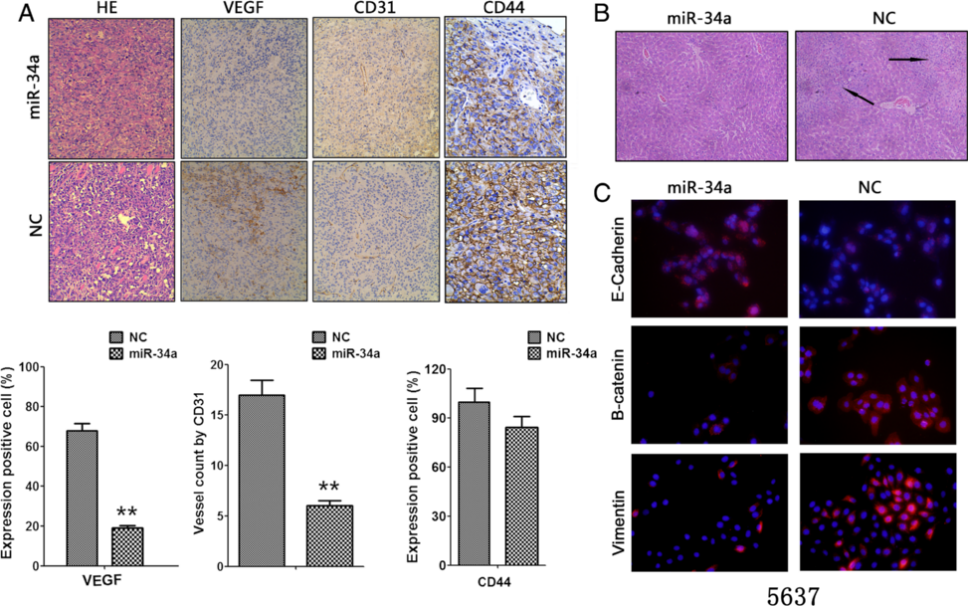
**Overexpression of miR-34a attenuated the metastasis and angiogenesis of bladder cancer in vivo. A**, hematoxylin/eosin (HE) and immunohistochemical staining revealed that stable transfection of miR-34a precursor resulted in decreased expression of VEGF, CD44 and CD31 within tumors. The mean vessel density within tumors decreased after stable overexpression of miR-34a. **B**, T24 cells were injected into the tail vein of nude mice (5 × 10^6^cells per mouse). Tumor cells stably infected with miR-34a lentivirus established significantly fewer metastatic colonies. **C**, Representative photographs of immunofluorescence were taken at × 400 magnification.

**Figure 4:**
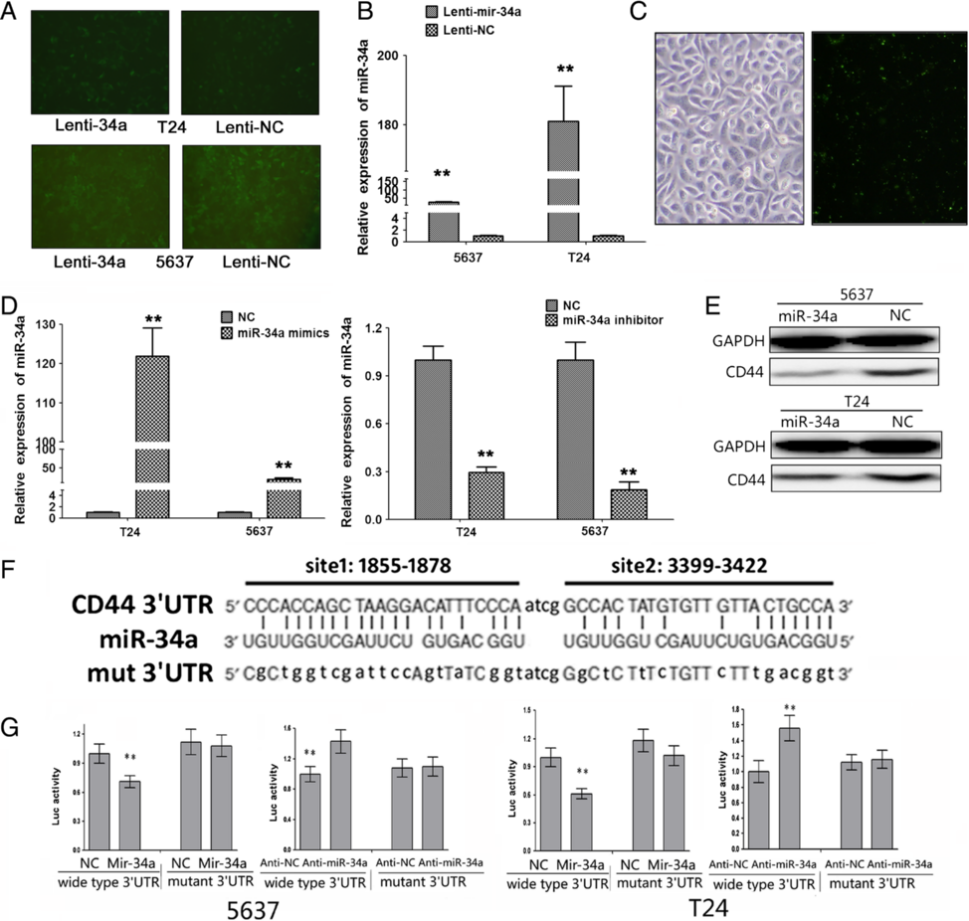
**mir-34a targets CD44 in bladder cancer cells and the validation of transfection/infection.** **A** and **B**, mir-34a expression was detected by qPCR after infection of miR-34a lentivirus and corresponding negative control in 5637 and T24 for 4 days. **C** and **D**, mir-34a expression was detected by qPCR after infection of miR-34a mimics or inhibitor and corresponding negative control in 5637 and T24 for 48 hours. CD44 protein expression was inhibited in miR-34a transfected bladder carcinoma cells. **E**, CD44 expression was detected by western blot after overexpression of miR-34a and corresponding negative control i 5637 and T24. **F** and **G**, The seed regions of the miR-34a target sites in CD44 and the luciferase activity assay. *, P < 0.05; **, P < 0.01.

To assess changes in cell migration, 5637 and T24 cells transfected with hsa-miR-34a mimics or FAM-labeled pre-miR-NC (negative control) were allowed to migrate through a transwell membrane into complete media. Compared with the negative control, mir-34a overexpression led to 55% and 65% reduction of migratory cells in 5637 and T24, respectively (Figure 2A).Then to evaluate cell invasion capability, hsa-miR-34a transfected 5637 and T24 cells were plated on the membrane which precoated with matrigel. As shown in Figure 2A, mir-34a overexpression significantly reduced 5637 and T24 cell invasion by approximately 32% and 56%. In intravenous injection assay, bioluminescence imaging revealed that fluorescence signal in lenti-mir-34a group were significantly weaker than lenti-NC group, which mean that less metastasis is formed in lung after mir-34a overexpression (Figure 2C). We also found that the number of metastatic nodules in the liver was dramatically decreased in miR-34a groups compared with negative controls (Figure 2G). Tumor cells stably infected with miR-34a lentivirus established significantly fewer metastatic colonies (Figure 3B). E-cadherin, β-catenin and Vimentin were also detected with immunofluorescence in 5637 cells. Result shown that miR-34a overexpression increased E-cadherin expression but decreased β-catenin and Vimentin expression (Figure 3C). Moreover, EMT-related genes like N-Cadherin, E-Cadherin, Vimentin and Beta-catenin were also examined by immunoblot and qPCR assay after mir-34a restoration. Result indicated that miR-34a could promote E-cadherin and inhibited N-cadherin, Beta-catenin and Vimentin mRNA in all these cell lines. Immunoblot showed that miR-34a consistently promoted E-cadherin protein expression and inhibited N-cadherin, Beta-catenin and Vimentin protein expression in all these cell lines. These results suggested that miR-34a could inhibit EMT in bladder cancer cell (Figure 2I, Figure 2J and Figure 4A). Some kinds of EMT-driving transcription factors are responsible for EMT phenomenon in this research article (Figure 4G). The data indicated that miR-34a functions as a tumor suppressor in bladder cancer.

### Mir-34a suppressed the angiogenesis of bladder cancer cells in vivo and vitro

Some previous studies indicate that CD44 promotes the invasion and angiogenesis of tumor cells [25,28]; we further investigated the effects of miR-34a overexpression and CD44 restoration on cultured bladder cancer cells. Western blotting indicated that transfection of CD44 rescued the miR-34a–induced downregulation of CD44 (Figure 4B). The tube formation of endothelial cells was suppressed by treatment with the medium preconditioned by stable transfection of bladder cancer cells with miR-34a precursor (Figure 2E). We next investigated the efficacy of miR-34a against angiogenesis in vivo. In the xenograft tumors, the VEGF and CD44 were also reduced by stable transfection of miR-34a precursor (Figure 3A). Moreover, stable transfection of miR-34a precursor resulted in a decrease in CD31-positive mean vessel density within tumors (Figure 3A). These results indicated that miR-34a remarkably decreased the angiogenesis of bladder cancer cells in vivo and vitro.

### Knockdown of CD44 suppressed the migration, invasion and angiogenesis of bladder cancer cells

**Figure 5:**
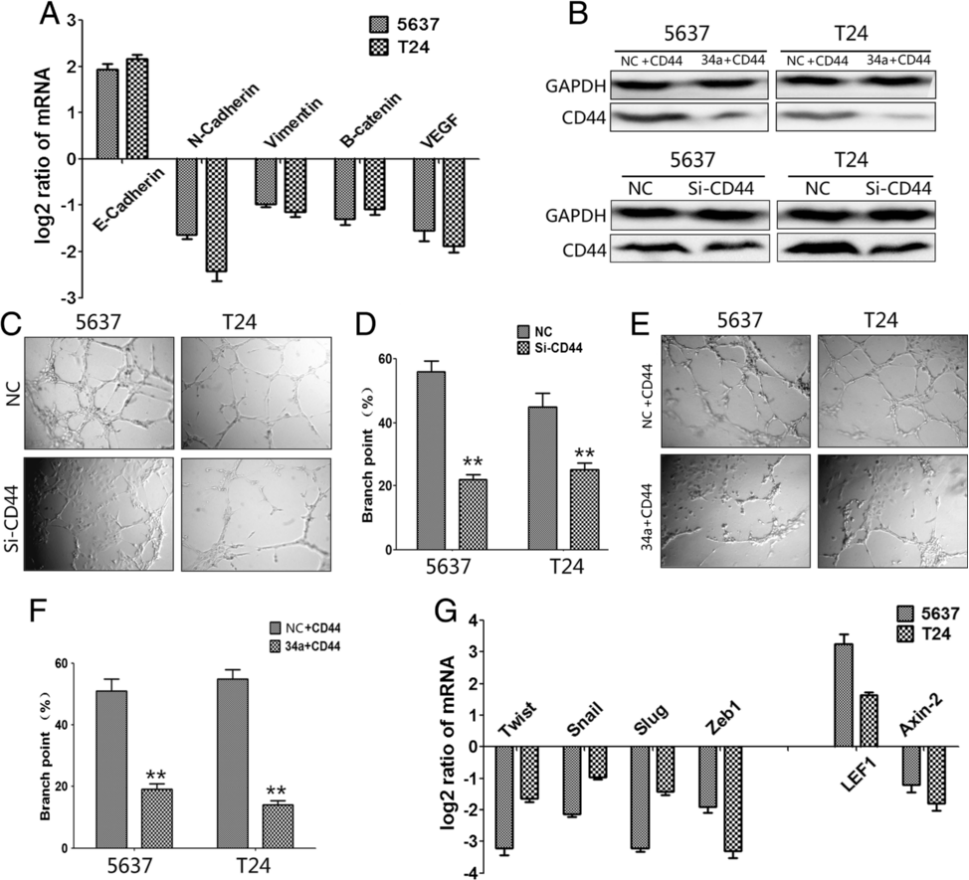
**The anti-angiogenesis functions of miR-34a were mediated by reducing the production of CD44.** Knockdown of CD44 by siRNA inhibits tube-formation and increased CD44 expression could efficiently reverse the effect of anti-angiogenesis of miR-34a in bladder cancer cells **A**, EMT related factors and VEGF was detected by qPCR. Data are plotted as the mean ± SEM of 3 independent experiments. **B**, western blotting indicated that transfection of si-CD44, into 5637 and T24 cells resulted in decreased CD44 expression when compared with negative control. Western blotting indicated that transfection of CD44 restored the downregulation of CD44 induced by stable miR-34a overexpression. **C** and **D**,the tube formation of endothelial HUVEC cells was suppressed by treatment with the medium preconditioned by CD44-knockdown 5637 and T24 cells, when compared with that of control cells. **E** and **F**, increased CD44 expression could efficiently reverse the effect of anti-angiogenesis of miR-34a in bladder cancer cells. *, P < 0.05; **, P < 0.01. **G**, exchange of EMT-driving transcription factors, LEF1 and Axin-2 in T24/5637-mir-34s.

Because above results indicated the negative regulation of CD44 expression by miR-34a, we hypothesized that knockdown of CD44 should have a similar effect on cultured bladder cancer cells. To this end, 5637 and T24 cells were infected with lentiviral constructs containing siRNA against CD44 or the negative control (Figure 4B). Remarkably, silencing of CD44 inhibited 5637 and T24 cells angiogenesis (Figure 4C), migration and invasion (Figure 5A). These results were consistent with the findings that overexpression of mir-34a suppressed the angiogenesis, migration and invasion of bladder cancer cells in vitro, providing further evidence that CD44 was involved in mir-34ameiated suppression of bladder cancer cells. Accordingly, identification of CD44 as a miR-34a target gene may explain, at least in part, why overexpression of miR-34a suppressed the migration, invasion, and angiogenesis of bladder cancer cells.

### CD44 was a candidate target of mir-34a and mir-34a interacted with a putative binding site in the CD44 3’UTR

**Figure 6:**
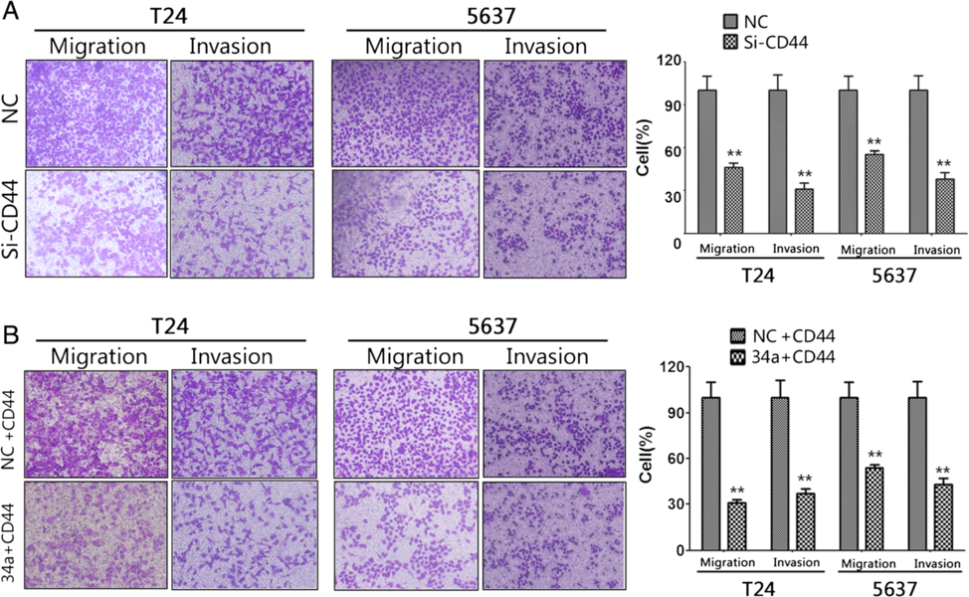
**The anti-metastatic functions of miR-34a were mediated by reducing the production of CD44.** Knockdown of CD44 by siRNA inhibits bladder cancer migration and invasion. Increased CD44 expression could efficiently reverse the effect of anti-metastatic of miR-34a in bladder cancer cells. **A**, matrigel invasion assay indicated the decreased invasion capabilities of CD44-knockdown 5637 and T24 cells than those of control cells. **B**, Transfection of CD44 rescued the angiogenic capabilities of miR-34a-overexpressing cells. *, P < 0.05; **, P < 0.01.

To investigate the hypothesis that miR-34a may influence the CD44 expression in bladder cancer, combination with the previous reports, computational prediction was done by miRNA databases. In the CD44 3’-UTR, there was one potential binding site of miR-34a with high complementarity (Figure 6F), the miR-34a- binding site was located at bases 1855–1878 and 3399–3422 of the CD44 3’-UTR, respectively (Figure 6F). Next, we investigated the direct effects of miR-34a on CD44 expression in bladder cancer cell lines; we conducted the miRNA overexpression experiments. Overexpression of miR-34a precursor into bladder cells resulted in increase of miR-34a levels (Figure 6A-D). Then western blotting, showed that stable transfection of miR-34a precursor resulted in decreased protein levels of CD44 in bladder cancer cells, when compared with negative control (Figure 6E). To determine whether miR-34a could repress CD44 expression or not by targeting its binding sites in the CD44 3’-UTR, the PCR products containing intact target sites or a mutation of miR-34a seed recognition sequence (Figure 6F) were inserted into the luciferase reporter vector. The plasmids were transfected into bladder cancer cells stably transfected with empty vector or miR-34a precursor. The luciferase activity normalized to that of firefly was significantly reduced in the tumor cells stably transfected with miR-34a precursor (Figure 6G), and the effect was abolished by mutating the putative miR-34a–binding site within the 3’-UTR of CD44 (Figure 6G). Moreover, knockdown of miR-34a with anti-miR-34a inhibitor increased the luciferase activity in 5637 and T24 cells (Figure 6G), whereas mutation of miR-34a recognition site abolished these effects (Figure 6G). These results indicated that miR-34a directly and specifically interacted with the target site in the CD44’-UTR.

### MiR-34a function as an anti-metastatic microRNA via directly targeting CD44 in bladder cancer

CD44 was overexpressed in bladder cancer and had a central role in regulating diverse aspects of bladder cancer pathogenesis. Interestingly, as predicated by TargetScan and previous study, miR-34a could negatively regulate different kinds of mRNAs including CD44 in some human cancers. Restoration of miR-34a could markedly decrease CD44 protein (Figure 6E). According to the previous study in prostate cancer [30] dual luciferase reporter assay was used and determined CD44 was also exactly the target of miR-34a in bladder cancer cells (Figure 6G). To confirm the synergistic effect of CD44 and miR-34a in tumorigenesis, we pulled down the expression of CD44 by specific siRNA resulting in decreased capability of cell angiogenesis, invasion and migration (Figure 4C and Figure 5A), which is in parallel with miR-34a overexpression in all the bladder cancer cell lines. Subsequently, we proved that restoration of CD44 could rescue the antitumor effects of miR-34a by co-transfection of miR-34a mimics and CD44 in bladder cancer cell lines (Figure 4E and Figure 5B). All data presented here strongly support our hypothesis that the tumor-suppressive effect of miR-34a is mediated by directly targeting CD44.

## Discussion

Over the past decade, the dysregulation of miRNAs contributed to the pathogenesis of most human cancers, and some novel researches supported that miRNAs were acting as activators or suppressors of metastasis and angiogenesis [43]. In our study, we identified a crucial tumor-suppressive miRNA, mir-34a, which plays an important role in the progression in bladder cancer. Some important prior studies including ours have reported on the dysregulation of various miRNAs including mir-34a in bladder cancer [22,25,27]. Although there are many related research, that microRNA-34a functions as an anti-metastatic microRNA and suppresses angiogenesis in bladder cancer by directly targeting CD44 still need further illuminated. Besides, although several other studies have investigated circulating miRNAs, they demonstrated that mir-34a plays a key role in NAFLD and colorectal cancer pathogenesis is supported by the evidence that serum levels of circulating mir-34a increased in participants with NAFLD and colorectal cancer, and they put forward that mir-34a may present a therapeutic target for NAFLD and colorectal cancer treatments, and a novel biomarker to predict NAFLD and colorectal cancer susceptibility and progression [44,45]. There have few reports on the expression of circulating mir-34a in bladder cancer, and the level of miR-34a was correlated with the level of mir-34a in bladder cancer tissues. An interesting study has suggested that a panel of miRNAs may have greater sensitivity and specificity than use of any single miRNA in identifying diseases [46,47]. Now, they alert us that it is possible that miR-34acombined with another miRNA, mRNAs or LncRNAs as part of a “biomarker panel” may together provide sufficient sensitivity and specificity to distinguish bladder cancer cases from controls and from other types of malignancies. Although further work needs to be done in order to clarify this, our next work will focus on this subject. These data indicated that the circulating level of mir-34a is proportionately associated with bladder cancer mir34a expression and circulating miRNAs may be able to be used as biomarker for bladder cancer.

Mir-34a has been reported taken great part in the occurrence and development of some human cancer contains bladder cancer [25], prostate cancer [30], cervical carcinoma [48], renal cancer [49] by targeting certain targets. And previous studies reported that upregulated the expression of miR-34a could exchange the expression of beta-catenin [41,50]. Our studies focus on it that microRNA-34a functions as an anti-metastatic microRNA and suppresses angiogenesis in bladder cancer by directly targeting CD44 could further enrich the theory of biology. Here, on the basis of our functional tests, findings that miR-34a was frequently downregulated in bladder cancer tissues and that miR-34a could suppress cell migration and invasion, and suppress tubefomation. Our data in vivo suggested that altered expression of miR-34a played a role in bladder cancer cellular metastasis and angiogenesis. In the subsequent mechanism research, mir-34a has many different certain targets in regulating different kinds of human cancer. CD44 overexpression eliminated the pro-apoptotic effects of miR-34a which is similar to what’s observed in medulloblastoma [51]. CD44 has been identified to be the direct target of miR-34a in prostate cancer, bladder cancer and renal cancer [25,28,30]. These results suggest that the role of miR-34a is possibly tumor-specific and highly dependent on its targets in different cancer cells. Our study proved that mir-34a functions as an anti-metastatic microRNA and suppresses angiogenesis by directly targeting CD44 in bladder cancer and subsequently suppress CD44 that regulates transcription of a variety of genes in bladder cancer cells.

## Conclusions

In summary, our research has demonstrated that miR-34a is significantly downregulated in bladder cancer tissues and bloodstream. MiR-34a overexpression can inhibit bladder cell migration, invasion, tubefomation in vitro and metastasis and angiogenesis in vivo. Furthermore, CD44 is a direct and functional target of miR-34a, and the functions that CD44-mediated can be reversed by miR-34a in bladder cells. This novel miR-34a/CD44/EMT related factor provides new insight into the mechanisms underlying tumor metastasis in bladder cancer. Combination with other pales of new specific biomarkers and restoration of miR-34a expression may be a potential diagnostic and therapeutic strategy to assess the prognosis and treatment of bladder cancer in the future.
